# Platelet-derived growth factor (PDGF) cross-signaling via non-corresponding receptors indicates bypassed signaling in colorectal cancer

**DOI:** 10.18632/oncotarget.28281

**Published:** 2022-10-19

**Authors:** Romana Moench, Martin Gasser, Karol Nawalaniec, Tanja Grimmig, Amrendra K. Ajay, Larissa Camila Ribeiro de Souza, Minghua Cao, Yueming Luo, Petra Hoegger, Carmen M. Ribas, Jurandir M. Ribas-Filho, Osvaldo Malafaia, Reinhard Lissner, Li-Li Hsiao, Ana Maria Waaga-Gasser

**Affiliations:** ^1^Renal Division, Brigham and Women’s Hospital, Harvard Medical School, Boston, MA 02115, USA; ^2^Department of Surgery I, Molecular Oncology and Immunology, University of Wuerzburg, Wuerzburg 97080, Bavaria, Germany; ^3^Department of Surgery I, University of Wuerzburg, Wuerzburg 97080, Bavaria, Germany; ^4^Shenzhen Traditional Chinese Medicine Hospital, Shenzhen 518033, Guangdong Province, China; ^5^Institute for Pharmacy and Food Chemistry, University of Wuerzburg, Wuerzburg 97074, Bavaria, Germany; ^6^Mackenzie Evangelical Faculty of Paraná, Curitiba 80730-000, Parana, Brazil; ^*^Co-senior investigators

**Keywords:** PDGF, VEGFR, EGFR, bypassed signaling, colorectal cancer

## Abstract

Platelet-derived growth factor (PDGF) signaling, besides other growth factor-mediated signaling pathways like vascular endothelial growth factor (VEGF) and epidermal growth factor (EGF), seems to play a crucial role in tumor development and progression. We have recently provided evidence for upregulation of PDGF expression in UICC stage I–IV primary colorectal cancer (CRC) and demonstrated PDGF-mediated induction of PI3K/Akt/mTOR signaling in CRC cell lines. The present study sought to follow up on our previous findings and explore the alternative receptor cross-binding potential of PDGF in CRC. Our analysis of primary human colon tumor samples demonstrated upregulation of the PDGFRβ, VEGFR1, and VEGFR2 genes in UICC stage I-III tumors. Immunohistological analysis revealed co-expression of PDGF and its putative cross-binding partners, VEGFR2 and EGFR. We then analyzed several CRC cell lines for PDGFRα, PDGFRβ, VEGFR1, and VEGFR2 protein expression and found these receptors to be variably expressed amongst the investigated cell lines. Interestingly, whereas Caco-2 and SW480 cells showed expression of all analyzed receptors, HT29 cells expressed only VEGFR1 and VEGFR2. However, stimulation of HT29 cells with PDGF resulted in upregulation of VEGFR1 and VEGFR2 expression despite the absence of PDGFR expression and mimicked the effect of VEGF stimulation. Moreover, PDGF recovered HT29 cell proliferation under simultaneous treatment with a VEGFR or EGFR inhibitor. Our results provide some of the first evidence for PDGF cross-signaling through alternative receptors in colorectal cancer and support anti-PDGF therapy as a combination strategy alongside VEGF and EGF targeting even in tumors lacking PDGFR expression.

## INTRODUCTION

As the third most common cancer, CRC exhibits one of the highest tumor-associated death rates in industrialized nations. Improvements in therapeutic options including chemoradiation protocols, an increasing number of approved antibodies for targeted therapies, and the development of small molecule therapeutics, have resulted in better tumor-related and overall survival in advanced stage patients. However, the overall 5-year survival rate remains only 65% and illustrates the need for new therapeutic strategies for patients with advanced stage cancer [1].

Multimodal CRC therapy usually comprises tumor resection and, if applicable in UICC (Union for International Cancer Control) stage III, treatment may be continued with chemotherapy with or without radiation integrated in a (neo-)adjuvant protocol before or after surgery for rectal cancers. Current chemotherapeutic treatment regimens for CRC consist of a combination of folinic acid, 5-fluorouracil (5-FU), and oxaliplatin (FOLFOX) or folinic acid, 5-FU, and irinotecan (FOLFIRI) [2, 3]. In recent years, new insights into the underlying mechanisms of angiogenesis and the growth factors necessary for tumor growth and overall progression have led to new treatment strategies in metastatic colorectal cancer (UICC stage IV). The vascular endothelial growth factor (VEGF) [4, 5] and its receptors as well as the epidermal growth factor (EGF) and its corresponding receptors [6, 7] have been intensively screened as potential therapeutic targets for the treatment of metastatic tumor growth. Monoclonal antibodies and tyrosine kinase inhibitors constitute new, moderately successful therapeutic options for UICC stage IV colorectal cancer patients and target VEGF and EGF signaling [8]. Numerous therapeutics have already been approved for CRC treatment including the EGFR inhibitors Cetuximab and panitumumab [6, 9–12], the VEGF-A inhibitors bevacizumab and aflibercept [4, 5, 13], and the VEGFR2 inhibitors regorafenib and ramucirumab [8, 14, 15]. Inhibition of VEGF, EGF, and their corresponding receptors decreases the activity of subsequent pathways (e.g., PI3K/Akt/mTOR) and mitigates cell proliferation and metabolism [16]. The monoclonal antibody, bevacizumab, neutralizes VEGF before it binds to its receptors and thus prevents the activation of oncogenic intracellular signaling pathways. Unfortunately, the efficacy of this targeted approach is impaired by the increased occurrence of resistance against this therapy. Even combined treatment against multiple different targets often lacks further benefit in clinical studies [16, 17].

**Figure 1:**
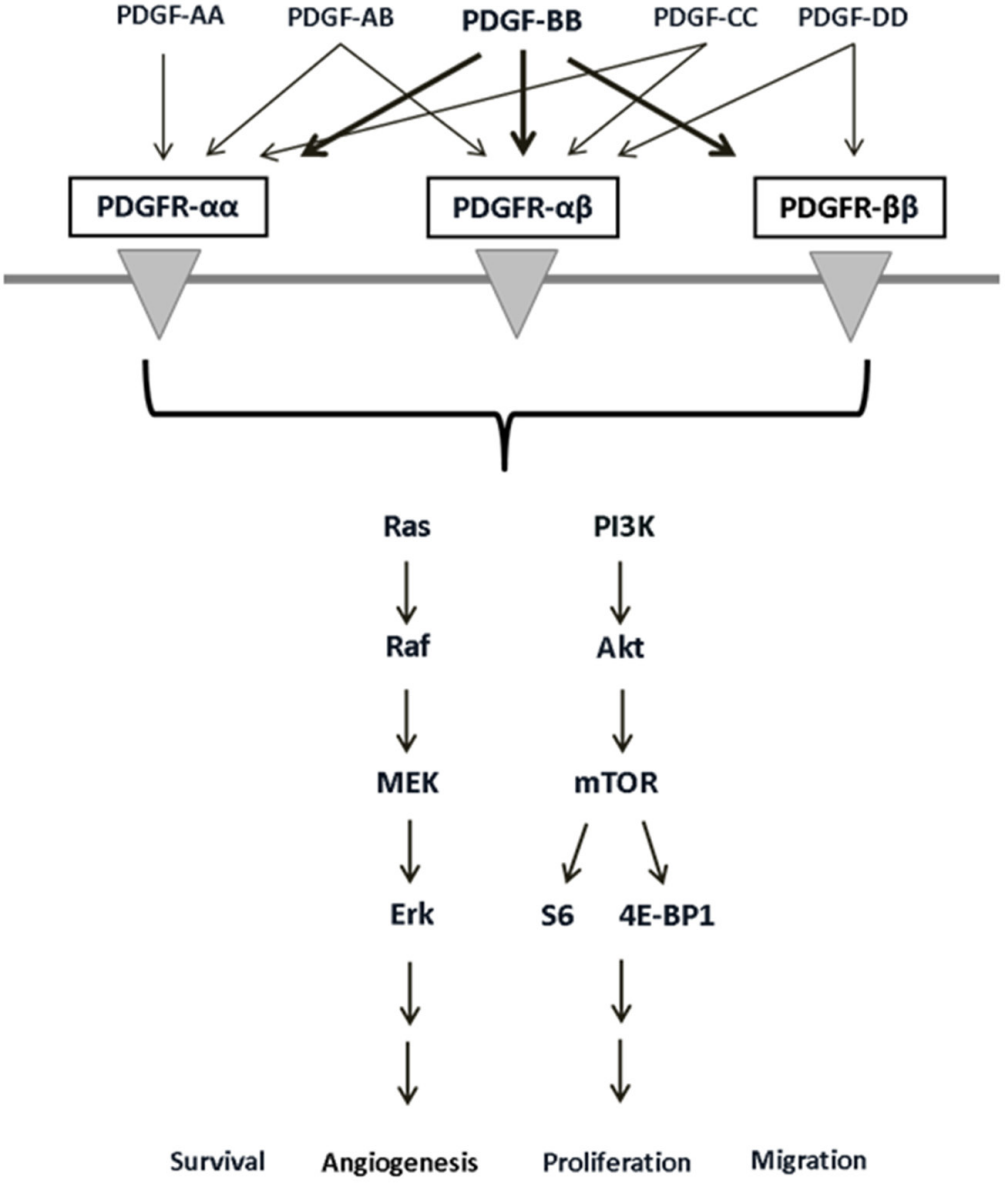
PDGF receptors, their binding ligands, and downstream signaling pathways with diverse cellular effects. PDGF receptor/ligand binding can activate the PI3K/Akt and MAPK pathways and influences cell survival, angiogenesis, proliferation, and migration.

The most important signaling pathways in CRC are regarded to be PI3K/Akt/mTOR and MAPK, both of which exert versatile effects on tumor cells [18–21]. The platelet-derived growth factor (PDGF), like VEGF, activates the phosphoinositide 3-kinase (PI3K/Akt/mTOR) and mitogen-activated protein kinase (MAPK) pathways [22–24]. These key signaling pathways mediate proliferation, migration, differentiation, and cell survival [22–25]. The PDGF family consists of 5 isoforms (PDGF-AA, -AB, -BB, -CC, -DD) that have the ability to bind to the PDGF receptor forms, alpha (α) and beta (β), with varying affinity [18]. PDGF-BB is a mitogen that is compatible with both PDGF-receptor forms [19] and plays a vital role in embryonic development, migration, organogenesis, and angiogenesis [26, 27]. PDGF-BB binds two receptors simultaneously and induces the dimerization and autophosphorylation thereof. The resulting transduction of intracellular signaling events exerts pro-oncogenic effects (Figure 1). PDGF and its receptors are expressed in colon cancer cells as well as surrounding tissue [26, 28, 29], implying the possibility of autocrine PDGF stimulation in tumor cells, which can boost the supportive effects of PDGF on tumor cells [19, 30]. We have recently provided evidence for overexpression of PDGF in CRC and demonstrated PDGF-mediated induction of cell signaling and proliferation in CRC cell lines [19]. Recent studies have likewise indicated the importance of PDGF for tumor development and its association with CRC progression and poor prognosis [19, 31], but the underlying molecular binding behavior of PDGF in CRC cells remains to be fully understood. Some studies have already provided evidence for PDGF binding to alternative receptors like VEGFR [32]. The present study analyzed PDGFR and VEGFR expression in primary colon cancer tissues and CRC cell lines in order to better define their expression profiles. Furthermore, we analyzed putative PDGF cross-binding with VEGFR and EGFR in HT29 cells lacking PDGFR expression.

## RESULTS

### PDGFRβ, VEGFR1, and VEGFR2 are overexpressed in human colon cancer

**Figure 2:**
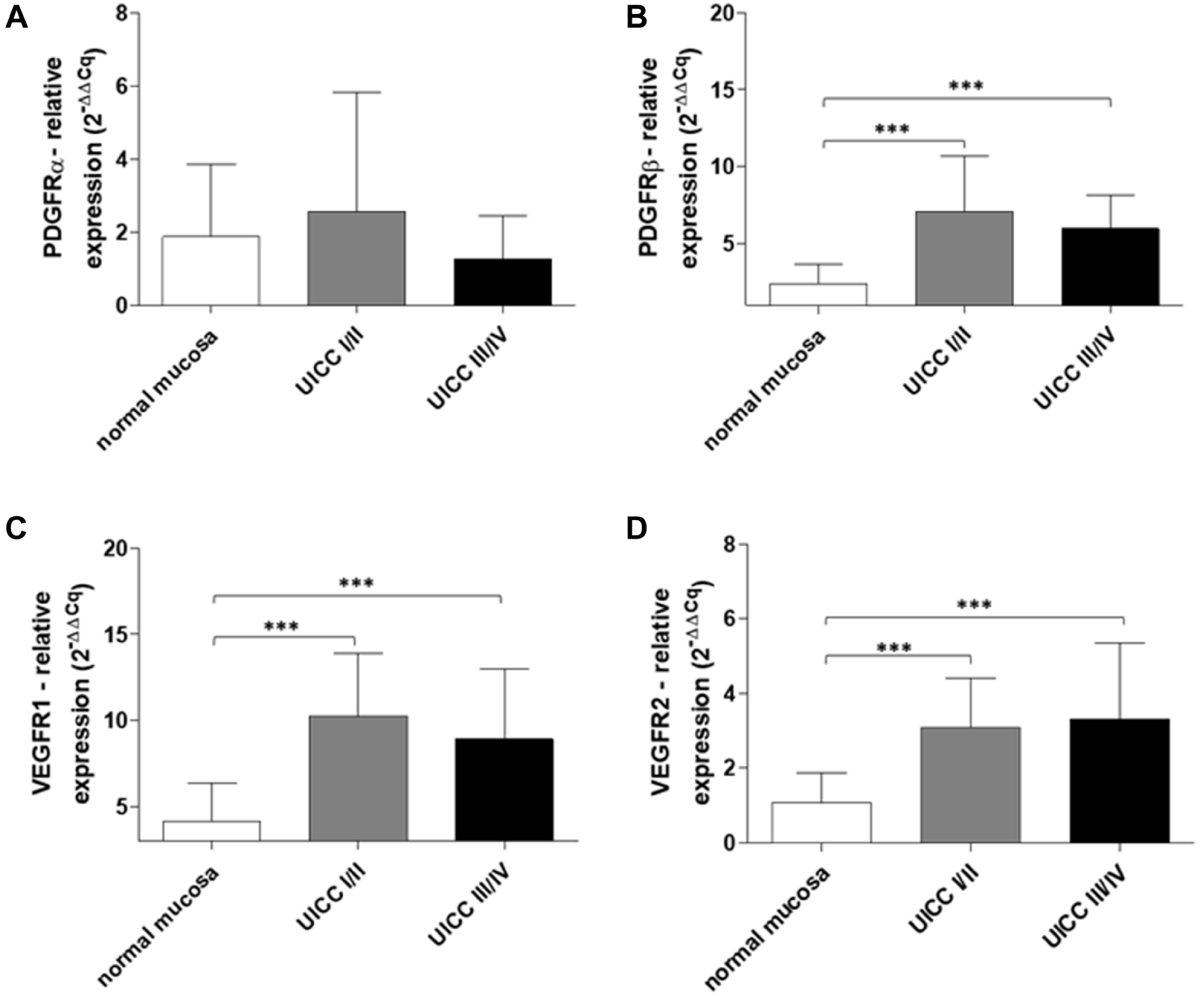
Increased PDGFR and VEGFR gene expression in early and advanced stage human colon cancer. RT-qPCR analysis of (**A**) PDGFRα, (**B**) PDGFβ, (**C**) VEGFR1, and (**D**) VEGFR2 in normal mucosa and human colon cancers (UICC stage I/II, *n* = 20 and UICC stage III/IV, *n* = 22). The relative quantification value is expressed as 2^−ΔΔCq^. Results are presented as mean ± SD; ^***^ *p* < 0.001.

To follow up on our previous finding of PDGF overexpression in CRC, we analyzed the expression of its corresponding receptors, PDGFRα and PDGFRβ, and presumed cross-binding partners, VEGFR1 and VEGFR2, in primary human colon cancer tissue samples (*n* = 42). Patient samples were divided into two groups representing early (UICC stage I/II) and advanced (UICC stage III/IV) stage tumors. Our results indicated no significant difference in the expression of PDGFRα in UICC stage I–IV tumors (Figure 2A). However, the expression of PDGFRβ, VEGFR1, and VEGFR2 was found to be significantly higher in UICC stage I-IV tumors than in normal mucosa (UICC stage I/II and III/IV versus normal mucosa: *p* < 0.001; Figure 2B–2D).

### PDGF is co-expressed with VEGFR2 and EGFR in human colon cancer

**Figure 3:**
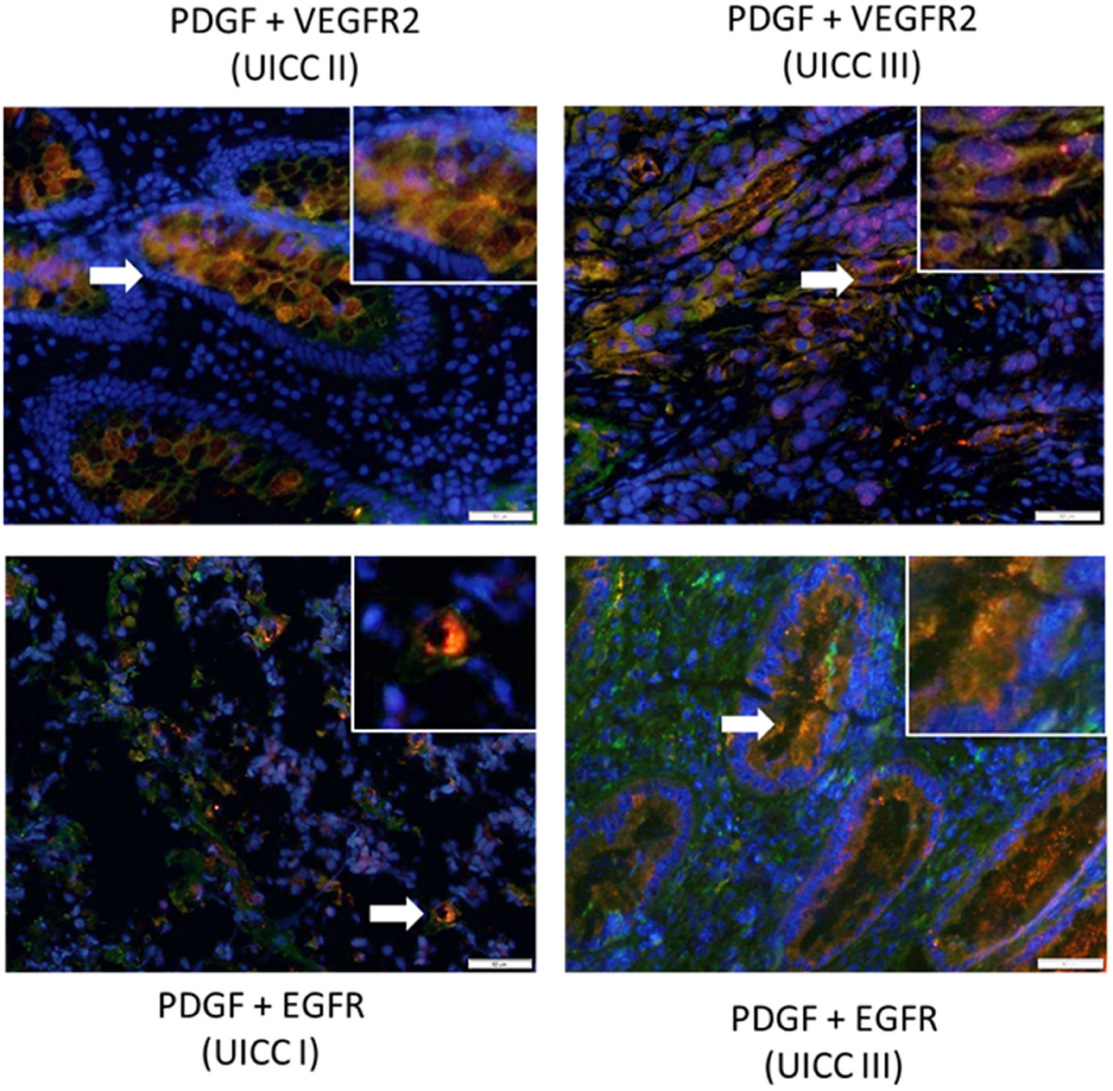
Co-expression of PDGF with VEGFR2 and EGFR in human colon cancer. Representative immunofluorescence double staining of colon cancer tissue (20 patient tissues were stained) exhibiting positive co-expression of PDGF (Cy3, red) and VEGFR2 or EGFR (Alexa 488, green). Arrows indicate regions demonstrating positive dual expression. Magnification 200×. Scale Bar = 50 uM.

To explore the possibility of PDGF cross-signaling via alternative receptors in CRC, we conducted a co-immunofluorescence staining analysis of PDGF and VEGFR2 or EGFR expression in primary human colon cancer tissue samples (*n* = 20). Our results demonstrate positive co-expression of PDGF and VEGFR2 or EGFR in tumor tissues from early (UICC stage I and II) and advanced (UICC stage III) stage patients (PDGF: Cy3, red; VEGFR2 and EGFR: Alexa 488, green; Figure 3).

### PDGFRα, PDGFRβ, VEGFR1, and VEGFR2 protein expression in colorectal cancer cells *in vitro*

**Figure 4:**
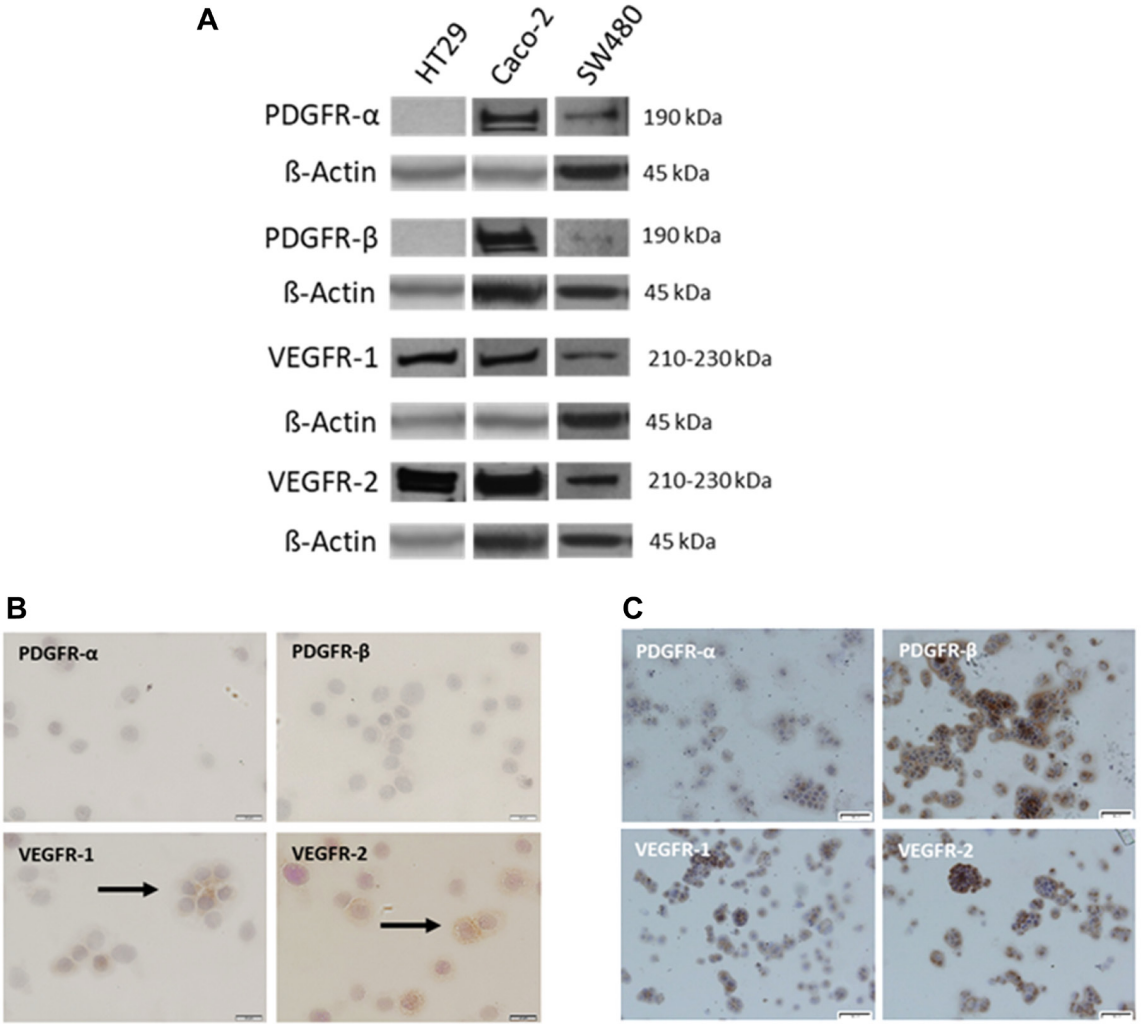
Variable expression of PDGFR and VEGFR in established CRC cell lines. (**A**) Representative Western blot analysis of PDGFRα, PDGFRβ, VEGFR1, and VEGFR2 in HT29, Caco-2, and SW480 CRC cells. β-Actin was used as loading control. Representative immunostaining of PDGFRα, PDGFRβ, VEGFR1, and VEGFR2 in (**B**) HT29 and (**C**) Caco-2 cells. Magnification: 200×. Scale Bar = 20 uM.

Given the observed overexpression of PDGF receptors and VEGF receptors in human colon cancer tumors, we assessed the protein expression of PDGFRα, PDGFRβ, VEGFR1, and VEGFR2 in established human colorectal cancer cell lines ranging from more differentiated and less metastatic HT29 and Caco-2 cells to the poorly differentiated SW480 cells. Western blot analysis revealed variable protein expression of the analyzed receptors amongst the investigated cell lines. Caco-2 and SW480 cells both demonstrated positive expression of all analyzed receptors; however, expression of these receptors was highest in Caco-2 cells. Interestingly, HT29 cells only demonstrated expression of VEGFR1 and VEGFR2 (Figure 4A). Immunostaining confirmed the absence of PDGFRα/β and presence of VEGFR1/2 expression in HT29 cells (Figure 4B), as well as positive expression of all analyzed receptors in Caco-2 cells (Figure 4C).

### PDGF induced transcriptional activity of relevant growth factor receptors in colorectal cancer cell lines

**Figure 5:**
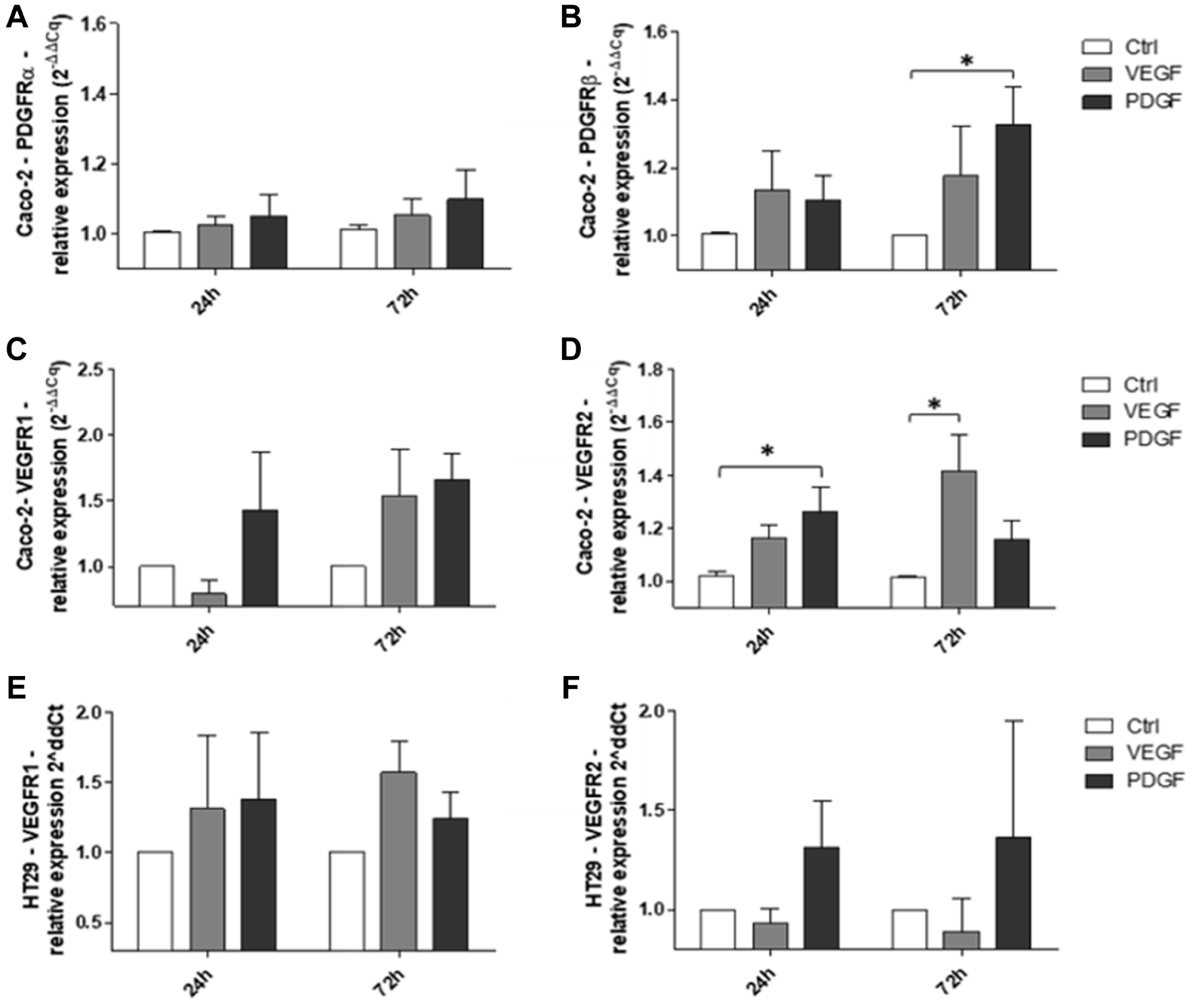
Effects of PDGF and VEGF on corresponding receptor expression in Caco-2 and HT29 CRC cells. RT-qPCR analysis of (**A**) PDGFRα, (**B**) PDGFRβ, (**C**) VEGFR1, and (**D**) VEGFR2 expression following stimulation of Caco-2 cells with PDGF or VEGF. RT-qPCR analysis of (**E**) VEGFR1 and (**F**) VEGFR2 expression following stimulation of HT29 cells with PDGF or VEGF. The relative quantification value is expressed as 2^−ΔΔCq^. Results are presented as mean ± SD, ^*^ *p* < 0.05, *n* = 3.

To provide insights into the presumed cross-signaling of PDGF via alternative growth factor receptors, we assessed PDGF-/VEGF-mediated effects on the transcriptional activity of their corresponding receptors in Caco-2 and HT29 colorectal cancer cell lines. In Caco-2 cells, stimulation with PDGF or VEGF resulted in only a slight increase in PDGFRα gene expression at both 24 and 72 hours (Figure 5A). Similar results were observed for PDGFRβ gene expression following stimulation with PDGF or VEGF at 24 hours; however, the expression of PDGFRβ was further enhanced at 72 hours, particularly following PDGF stimulation (PDGF versus Ctrl: *p* < 0.05; Figure 5B). Moreover, VEGFR1 expression was enhanced following stimulation with PDGF at 24 hours and either PDGF or VEGF at 72 hours (Figure 5C). Additionally, stimulation of Caco-2 cells with either PDGF or VEGF resulted in a significant increase in VEGFR2 expression at 24 and 72 hours, respectively (PDGF versus Ctrl at 24 hours: *p* < 0.05, VEGF versus Ctrl at 72 hours: *p* < 0.05; Figure 5D). Given the finding that PDGFRα and PDGFRβ were not expressed in HT29 cells, we sought to determine if PDGF could affect these cells despite the absence of its corresponding receptors. Interestingly, our results showed that stimulation of HT29 cells with PDGF or VEGF resulted in an increase in VEGFR1 expression at 24 and 72 hours (Figure 5E). Additionally, only PDGF affected VEGFR2 expression in these cells (Figure 5F). Together, the overall comparable effect of PDGF and VEGF to upregulate PDGF receptor and VEGF receptor expression, particularly in HT29 cells lacking PDGF receptor expression, suggest the possibility of an alternative receptor cross-binding signaling mechanism in CRC.

### PDGF promoted proliferation of HT29 colorectal cancer cells lacking PDGFR expression

**Figure 6:**
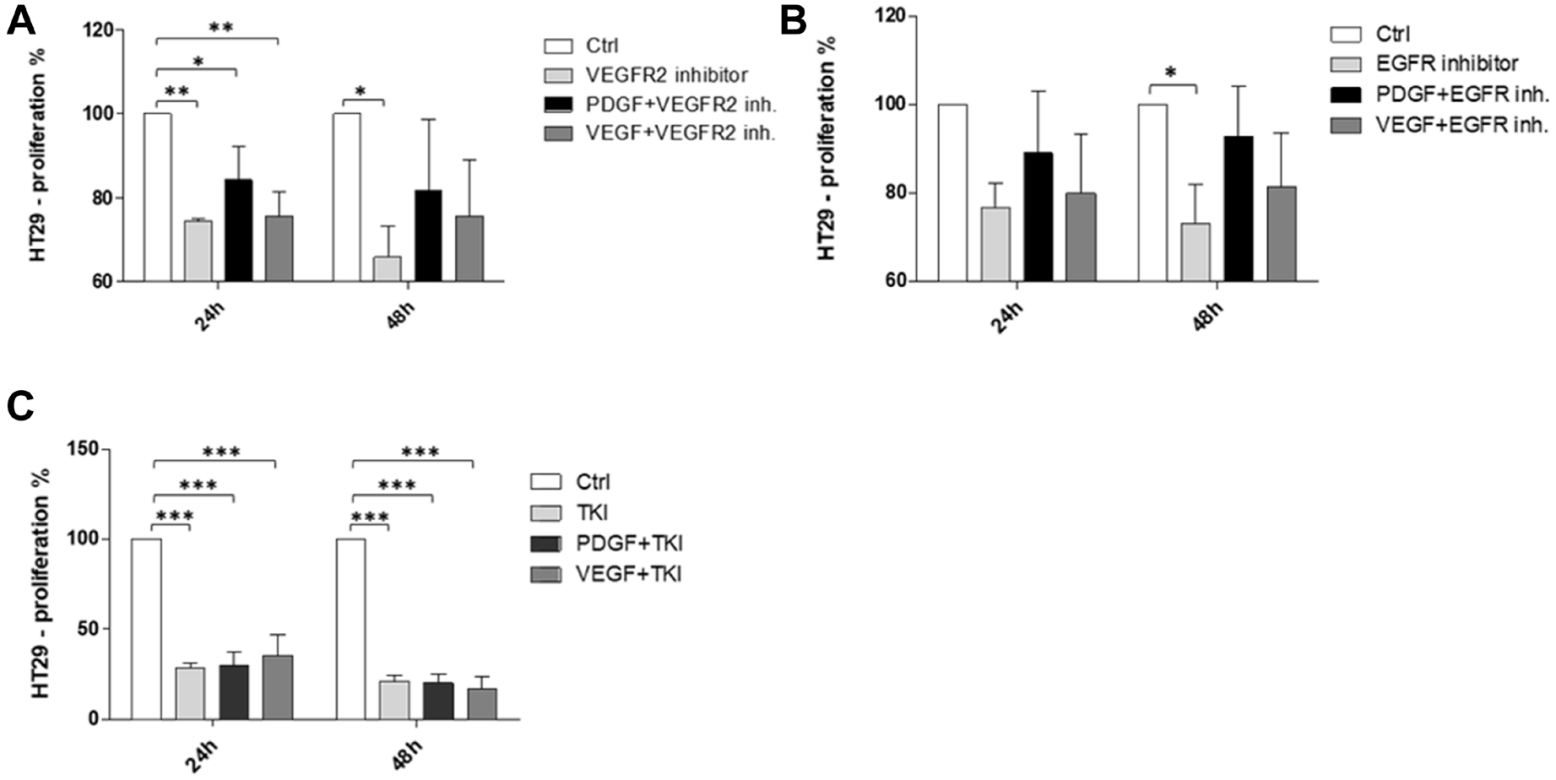
Pro-proliferative effects of PDGF on HT29 CRC cells under simultaneous inhibition of VEGFR2 or EGFR. PDGF recovered proliferation of cells treated with (**A**) the VEGFR2 inhibitor, ramucirumab, and (**B**) the EGFR inhibitor, cetuximab. Neither PDGF nor VEGF were able to recover proliferation of cells treated with (**C**) the broad range TKI, regorafenib. Results are presented as ± SD, ^*^ *p* < 0.05, ^**^ *p* < 0.01, ^***^ *p* < 0.001, *n* = 3.

The cross-binding potential of PDGF with alternative receptors was further investigated in HT29 cells by assessing the pro-proliferative effects of PDGF combined with simultaneous selective inhibition of relevant receptors. Our results showed that PDGF-mediated stimulation of cells treated with the VEGFR2 inhibitor, ramucirumab, resulted in a recovery of cell proliferation at 24 and 48 hours (Figure 6A). Moreover, PDGF also resulted in recovered proliferation of cells treated with the EGFR inhibitor, cetuximab, at 24 and 48 hours (Figure 6B). Unlike inhibition of VEGFR2 or EGFR, neither PDGF nor VEGF were able to recover HT29 cell proliferation during simultaneous treatment with the broad range tyrosine kinase inhibitor, regorafenib (Figure 6C).

## DISCUSSION

Recent findings have suggested that PDGF-mediated signaling in solid tumors plays a significant role in cell migration and intratumoral angiogenesis. Consequently, inhibition of the PDGF signaling pathway has come to the forefront of interest in the treatment of patients with advanced stage CRC [33–35]. Such targeting would follow the successfully implemented concept to inhibit VEGF-mediated angiogenesis in patients with metastasis. With further evidence for its significance, suppression of PDGF-mediated signaling in CRC may be a particularly valuable tool to improve limited survival in advanced stage disease individuals at risk for tumor recurrence. This may be administered as a combination therapy beside inhibition of VEGF-mediated angiogenesis. In clinical practice, it has become apparent that monoclonal antibodies against VEGF and its receptor, VEGFR2, are effective in patients with CRC-derived liver metastases although overall therapeutic efficacy is often limited due to the development of resistance against treatment. Despite research into resistance mechanisms (*e.g.,* anti-idiotypic antibodies and receptor shift), it is still not possible to decisively prevent resistance [36]. Although recent studies have emphasized the potent role of PDGF in CRC, its influence appears to be multifactorial and therefore needs to be further clarified [31, 37].

We have recently provided evidence for upregulation of PDGF expression in UICC stage I-IV primary CRC tumors and demonstrated PDGF-mediated induction of PI3K/Akt/mTOR signaling in CRC cell lines [19]. In the present study, we sought to follow up on our previous findings with additional evidence clarifying PDGF-mediated signaling in CRC. We demonstrated that the gene expression of PDGFRβ, VEGFR1, and VEGFR2 was significantly higher in our cohort of UICC stage I-IV colon cancer patients compared to normal colon mucosa. These results suggest that PDGF and VEGF signaling is prominent in CRC development and progression; however, since both early and advanced stage tumors exhibited similar expression levels, their upregulation does not appear to be stage-dependent. We also conducted an immunohistological expression analysis of PDGF and its putative cross-binding partners. Our results revealed co-expression of PDGF and the alternative growth factor receptors, VEGFR and EGFR. Specifically, PDGF was found to be co-expressed with VEGFR2 as well as EGFR in UICC stage I-III tumors. This suggests that PDGF may bind and induce signaling through alternative growth factor receptors. Previous study has indeed provided evidence that PDGF may bind VEGF receptors [32].

Given the observed overexpression of PDGF/VEGF receptors in human colon cancer tumors, we analyzed several CRC cell lines for the expression of PDGFRα, PDGFRβ, VEGFR1, and VEGFR2. Our results demonstrated variable expression of the analyzed receptors, suggesting that even though expression may not be stage-dependent as evidenced by our analysis of human CRC tissues, it may depend on individual tumor profiles. Of the investigated cell lines, Caco-2 and SW480 cells both demonstrated positive expression of all analyzed receptors. Interestingly, HT29 cells showed no detectable expression of PDGFRα and PDGFRβ; however, VEGFR1 and VEGFR2 were both expressed. Given the prior evidence for PDGF cross-binding [32], we sought to determine if HT29 cells lacking PDGF receptor expression could be affected by PDGF stimulation. First, we stimulated Caco-2 cells with PDGF and VEGF. Given that Caco-2 cells expressed both PDGF receptors and VEGF receptors, we expected that stimulation with the respective ligands would result in enhanced transcription of the corresponding receptor genes. Our results confirmed this postulation. Next, we analyzed the effect of PDGF and VEGF on receptor expression in HT29 cells. Despite the absence of PDGF receptor gene and protein expression in these cells [31, 38, 39], we previously demonstrated cell signaling and metabolic alterations following treatment of HT29 cells with PDGF [19]. Interestingly, in the present study we found that stimulation of HT29 cells with PDGF or VEGF resulted in enhanced expression of VEGFR1 and VEGFR2. Given that the corresponding receptors for PDGF are not expressed in these cells, this finding suggests that PDGF signaling occurred through alternative receptors, namely VEGFR1 or VEGFR2. Although a PI3KCA mutation has been found in HT29 cells [40] and could contribute to enhanced signaling downstream of PI3K and an upregulation of VEGFR1 and VEGFR2 expression, the observed upregulation of VEGFR1 and VEGFR2 following PDGF stimulation relative to the control group suggests that PDGF cross-signaling is at least in part responsible for these results.

To further elucidate our finding, we employed selective inhibition of VEGFR2 and EGFR and simultaneous stimulation with PDGF to demonstrate its putative cross-signaling effects. We analyzed cell proliferation following 24 and 48 hours of combination treatment with PDGF and the selective inhibitors. Our results revealed that PDGF-mediated stimulation of cells treated with the VEGFR2 inhibitor, ramucirumab, or the EGFR inhibitor, cetuximab, resulted in a recovery of cell proliferation. These findings suggest that PDGF may competitively bind these receptors and that PDGFR-targeting in CRC therapy may not be enough to suppress PDGF-mediated signaling. However, unlike VEGFR2 or EGFR inhibition, proliferation of cells treated with the broad-range tyrosine kinase inhibitor, regorafenib, could not be recovered with PDGF stimulation. This suggests that the broader target range of regorafenib exerts a more profound inhibitory effect [41]; however, this quality may also render the TKI more susceptible to developing resistance. Indeed, it has been shown that regorafenib is not able to evade the development of resistance during the course of therapy by inhibiting several tyrosine kinases [42, 43].

**Figure 7:**
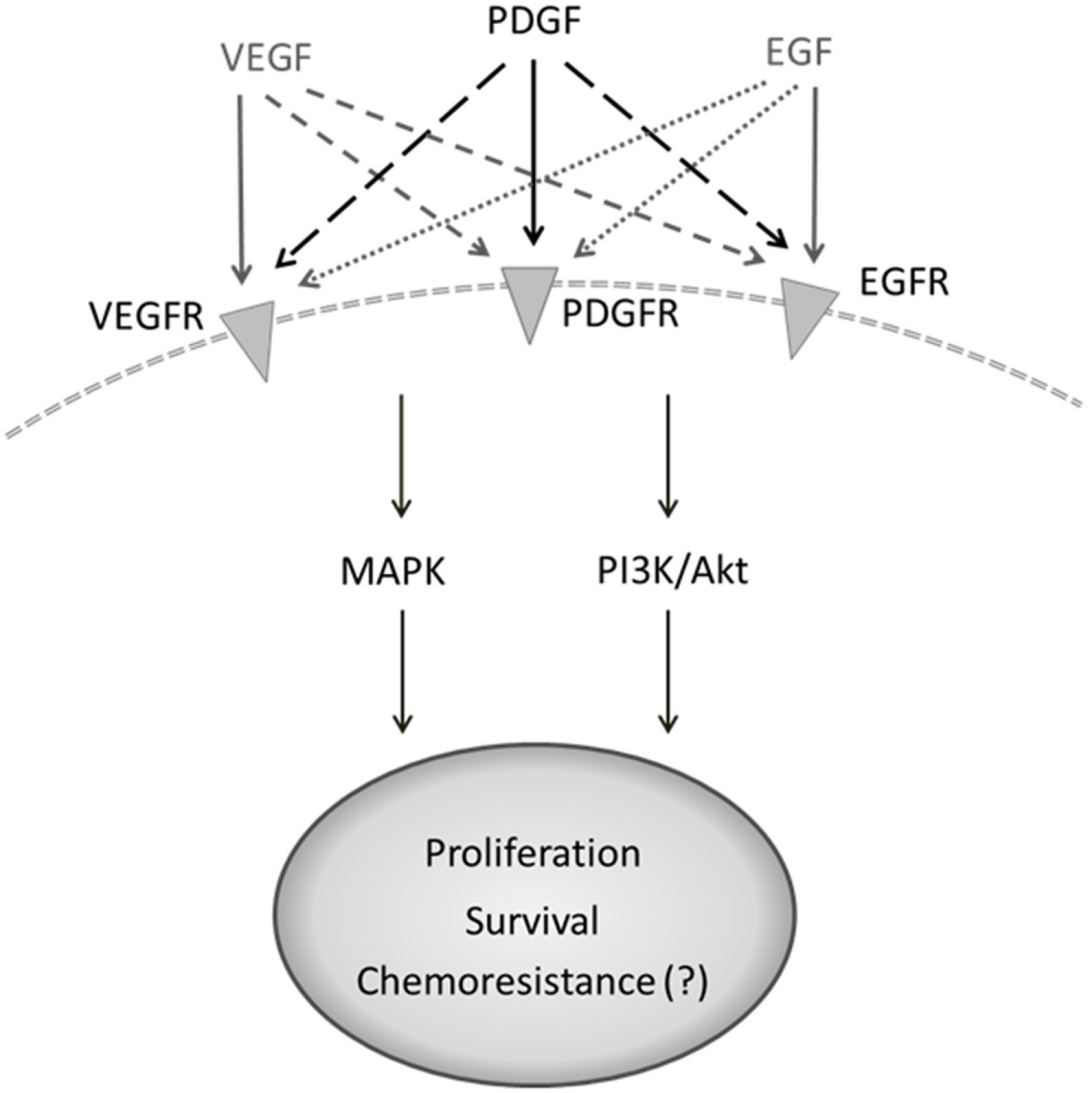
Suggested crosstalk of PDGF with alternative binding partners on tumor cell surface. Flexible binding of PDGF to alternative receptors like VEGFR or EGFR bypassing corresponding receptor signaling may result in ineffective outcome of therapies with monoclonal antibodies (anti-VEGFR or -EGFR) or tyrosine kinase inhibitors with limited target spectrum efficacy.

Altogether, our results indicate *in vitro* cross-signaling of PDGF via alternative tyrosine kinase receptors on the tumor cell surface beside solely PDGF receptors (Figure 7) and suggest that crosstalk of PDGF with related receptors may depend on the balance of several binding partners within each individual tumor. These findings offer relevance to the area of tumor cell resistance, further suggesting that PDGFR targeting in CRC treatment may not be enough to suppress PDGF-mediated effects on tumor development and progression. Future studies should focus on the particular interactions between PDGF and alternative receptors like VEGFR2 and EGFR so as to allow for the development of more precise and effective tyrosine kinase inhibitors and/or more comprehensive targeting regimens.

## MATERIALS AND METHODS

### Tissue samples

Tissue samples were obtained from 42 colon cancer patients (20 patients at UICC stage I/II, 22 patients at UICC stage III/IV) who underwent curative surgical resection in the Wuerzburg surgical department between 09/2009 and 05/2013. The study was conducted in accordance with the Declaration of Helsinki. Informed consent was obtained preoperatively. The Human Research Ethics Committee of the University of Wuerzburg gave ethical approval for this research.

### Human colorectal cancer cell lines

The human colorectal cancer cell lines HT29, Caco-2, and SW480 were obtained from ATCC (Manassas, VA, USA) and were tested for mycoplasma before experimental usage. Penicillin/streptomycin was purchased from Life Technologies (Carlsbad, CA, USA). Cells were cultured at 37°C in 5% CO_2_. SW480 and HT29 cells were cultured in RPMI and McCoy’s 5A media, respectively, supplemented with 10% (v/v) fetal bovine serum and 1% (v/v) penicillin/streptomycin. Caco-2 cells were cultured in Eagle’s Minimum Essential Medium supplemented with 20% (v/v) fetal bovine serum and 1% (v/v) penicillin/streptomycin.

### *In vitro* stimulation with PDGF and VEGF

PDGF-BB was obtained from Miltenyi Biotech (Bergisch Gladbach, Germany) and VEGF-165 from R&D Systems (Minneapolis, MN, USA). Cells were treated with 100 ng/ml PDGF or VEGF between 24 and 72 hours.

### Real-Time quantitative polymerase chain reaction

Gene expression of PDGFRα, PDGFRβ, VEGFR1, and VEGFR2 was analyzed by real-time quantitative polymerase chain reaction (RT-qPCR). An ImPromII reverse transcriptase system (Promega, WI, USA), and an Eppendorf Mastercycler (Eppendorf, Hamburg, Germany) were used to obtain complementary DNA (cDNA). TaqMan gene expression assays were purchased from Thermo Fisher Scientific (Waltham, MA, USA). All samples were assayed in duplicates and normalized during data analysis. β-Actin, 18 SrRNA, and RPLP0 (ribosomal protein lateral stalk subunit P0) were used as housekeeping genes for relative quantification. Tissue sample results were normalized to normal colon tissue (purchased from Biochain, Hayward, CA, USA). The relative quantification value is expressed as 2^−ΔΔCq^. PCR analysis was conducted with a BioRad CFX96 Touch real-time PCR detection system.

### Immunofluorescence staining

For immunofluorescence double staining, the colon cancer tissue slides were fixed in acetone and the antibodies bound during the first staining step were covered and fixed with Dako Doublestaining system, K1395 (Dako, Glostrup, Denmark), according to the manufacturer’s instructions. The following primary antibodies were used: PDGF (Santa Cruz Biotechnology), VEGFR2 (Abcam), and EGFR (Abcam). The following secondary antibodies were used: rabbit anti-mouse Cy3 and goat anti-rabbit Alexa 488 (Dianova, Hamburg, Germany). An Olympus BX51 microscope and the CellSens Dimension software were used for visualization.

### Protein extraction and Western blot analysis

Protein extracts were lysed in RIPA buffer (Life Technologies), electrophoresed using the NuPage system (Life Technologies), and transferred with the iBlot dry blotting system (Life Technologies). Proteins were detected with PDGFRα (D1E1E) antibody, PDGFRβ (28E1) antibody, VEGFR1 antibody, VEGFR2 (55B11) antibody (all purchased from Cell Signaling, Beverly, MA, USA), and with HRP-conjugated secondary antibody (Santa Cruz Biotechnology, Dallas, TX, USA). All antibodies were diluted to a concentration of 1:1000. Bands were detected by ECL solution (Thermo Fisher Scientific).

### Cytospin preparation and immunostaining

For cytospin preparations, HT29 or Caco-2 colorectal cancer cells were harvested using Accutase (Sigma-Aldrich, Munich, Germany) and adjusted to a final concentration of 2 × 10^5^ cells/ml. Cytospin preparations were performed with 50 μl of cell suspension at 550 rpm for one minute in a Cytospin4 cytocentrifuge (Thermo Fisher Scientific) and incubated with the following antibodies: PDGFRα (D1E1E) (Cell Signaling Technology) at a dilution of 1:500; PDGFRβ (28E1) (Cell Signaling Technology) at a dilution of 1:100; VEGFR1 (Y103) (Abcam, Cambridge, UK) at a dilution of 1:250; VEGFR2 (55B11) (Cell Signaling Technology) at a dilution of 1:200.

### Cell proliferation assay and receptor inhibition

Cell proliferation was detected with the colorimetric CellTiter 96 Aqueous One Solution assay (Promega, WI, USA). 2500 cells/well were seeded in a 96-well plate. Cells were washed twice with PBS and treated with 20 μg/ml ramucirumab (Cyramza^®^, Eli Lilly, Indianapolis, IN, USA), 1 mg/ml cetuximab (Erbitux^®^, Merck Serono, Darmstadt, Germany), or 10 μM regorafenib (AdipoGen, San Diego, USA), and stimulated with PDGF or VEGF (100 ng/ml respectively) for 24 hours and 48 hours under starving conditions. MTS proliferation assay was performed after 24 and 48 hours. CellTiter 96^®^ Aqueous One Solution was added to each well and measured according to the manufacturer’s instructions.

### Statistical analysis

Statistical analysis was performed using GraphPad Prism 5.0 (Graph Pad Software Inc., San Diego, CA, USA). A one-way ANOVA with Tukey’s post hoc test was used for the analysis of PDGFR and VEGFR gene expression in human tissues and CRC cell lines as well as the analysis of HT-29 cell proliferation. Data were presented as mean ± standard deviation. *p* < 0.05 was considered to be statistically significant.

## Abbreviations

CRC: colorectal cancer
EGFR: epidermal growth factor receptor
MAPK: mitogen-activated protein kinase
PDGFR: platelet-derived growth factor receptor
PI3K: phosphoinositide 3-kinase
TKI: tyrosine kinase inhibitor
UICC: Union for International Cancer Control
VEGFR: vascular endothelial growth factor receptor
